# Quantitative Proteomics Reveal That CB2R Agonist JWH-133 Downregulates NF-κB Activation, Oxidative Stress, and Lysosomal Exocytosis from HIV-Infected Macrophages

**DOI:** 10.3390/ijms25063246

**Published:** 2024-03-13

**Authors:** Lester J. Rosario-Rodríguez, Yadira M. Cantres-Rosario, Kelvin Carrasquillo-Carrión, Ana E. Rodríguez-De Jesús, Luz J. Cartagena-Isern, Luis A. García-Requena, Abiel Roche-Lima, Loyda M. Meléndez

**Affiliations:** 1 Department of Microbiology and Medical Zoology, University of Puerto Rico-Medical Sciences Campus, San Juan 00935, Puerto Rico; lester.rosario@upr.edu; 2 Translational Proteomics Center, Center for Collaborative Research in Health Disparities, University of Puerto Rico-Medical Sciences Campus, San Juan 00935, Puerto Rico; yadira.cantres@upr.edu (Y.M.C.-R.); ana.rodriguez48@upr.edu (A.E.R.-D.J.); 3 Integrated Informatics Core, Center for Collaborative Research in Health Disparities, University of Puerto Rico-Medical Sciences Campus, San Juan 00935, Puerto Rico; kelvin.carrasquillo@upr.edu (K.C.-C.); abiel.roche@upr.edu (A.R.-L.); 4 Department of Biology, University of Puerto Rico-Río Piedras Campus, San Juan 00925, Puerto Rico; luz.cartagena1@upr.edu (L.J.C.-I.); luis.garcia44@upr.edu (L.A.G.-R.)

**Keywords:** CATB, HIV, MDM, CB2R, NF-κB, Nrf2

## Abstract

HIV-associated neurocognitive disorders (HAND) affect 15–55% of HIV-positive patients and effective therapies are unavailable. HIV-infected monocyte-derived macrophages (MDM) invade the brain of these individuals, promoting neurotoxicity. We demonstrated an increased expression of cathepsin B (CATB), a lysosomal protease, in monocytes and post-mortem brain tissues of women with HAND. Increased CATB release from HIV-infected MDM leads to neurotoxicity, and their secretion is associated with NF-κB activation, oxidative stress, and lysosomal exocytosis. Cannabinoid receptor 2 (CB2R) agonist, JWH-133, decreases HIV-1 replication, CATB secretion, and neurotoxicity from HIV-infected MDM, but the mechanisms are not entirely understood. We hypothesized that HIV-1 infection upregulates the expression of proteins associated with oxidative stress and that a CB2R agonist could reverse these effects. MDM were isolated from healthy women donors (*n* = 3), infected with HIV-1_ADA_, and treated with JWH-133. After 13 days post-infection, cell lysates were labeled by Tandem Mass Tag (TMT) and analyzed by LC/MS/MS quantitative proteomics bioinformatics. While HIV-1 infection upregulated CATB, NF-κB signaling, Nrf2-mediated oxidative stress response, and lysosomal exocytosis, JWH-133 treatment downregulated the expression of the proteins involved in these pathways. Our results suggest that JWH-133 is a potential alternative therapy against HIV-induced neurotoxicity and warrant in vivo studies to test its potential against HAND.

## 1. Introduction

Approximately 15–55% of HIV-positive people develop despite being under combined antiretroviral therapy (cART) [1]. There are no effective therapies available against HAND. HAND is a neurodegenerative disease characterized by lymphocyte and MDM infiltration, inflammation, and persistent HIV replication in affected individuals’ brains [2,3,4,5,6]. Our laboratory has found increased expression of CATB, a proinflammatory lysosomal enzyme, in the post-mortem brain tissues, plasma, and monocytes of individuals with HAND [7,8]. Also, in in vitro HIV-infected macrophages, increased CATB release in its free-form leads to neuronal apoptosis [7,9,10,11,12,13,14]. We also demonstrated that CATB can be found free and in exosomes secreted from HIV-infected macrophages [15]. However, the mechanisms of free-form CATB secretion from HIV-infected macrophages are not entirely understood. In osteosarcoma cells, mitochondrial oxidative phosphorylation impairment and NF-κB (p65) activation lead to an increased expression and secretion of CATB [16]. IL-6 stimulation in osteoclasts promotes the release of CATB in part through NF-κB activation [17]. Similarly, in melanoma cells, NF-κB (p65) activation leads to CATB secretion [18]. Previous studies in our laboratory have proposed that the release of CATB to the extracellular space is associated with oxidative stress and NF-κB activation [19,20,21]. This was based on findings showing that the HIV-1 infection of macrophages decreased intracellular tyrosine-phosphorylated STAT-1 (STAT-1PY) levels due to STAT-1 interaction with cystatin B, sequestering STAT-1 in the cytoplasm and preventing its phosphorylation [22]. This sequestration may affect STAT1PY interaction with the interferon regulatory factor 1 (IRF-1), preventing IRF-1 binding with NF-κB and translocation to the nucleus [20]. Indeed, additional experiments with IFN-β-treated MDM demonstrated that cystatin B interferes with STAT-1 signaling and IFN-β–antiviral responses [21]. The activation of NF-κB may promote increased HIV-1 replication, oxidative stress, and the release of CATB into the extracellular space [20]. However, the mechanism by which CATB in its free-form could end up secreted from HIV-infected macrophages is unknown.

In previous studies, we demonstrated that increased CATB secretion from HIV-infected macrophages is associated with increased intracellular ROS/RNS levels, confirming our hypothesis that CATB secretion is prompted by oxidative stress [10]. The nuclear erythroid transcription factor 2 (Nrf2) is considered the master regulator of the oxidative stress response by activating the antioxidant response element (ARE) [23,24,25]. Studies have demonstrated that HIV-1 infection suppresses the Nrf2/ARE pathway [26,27,28,29]; however, cells exposed to HIV-1 viral proteins such as Tat, gp120, and the reverse transcriptase (RT) show discrepant effects [27,28,29,30,31,32,33]. Our previous study also demonstrated that dimethyl fumarate (DMF), a known NF-κB inhibitor and Nrf2 activator, decreases HIV-1 replication, oxidative stress, and CATB release from HIV-infected MDM, indicating that oxidative stress is linked to CATB secretion [10,34].

Subtle increases in reactive oxygen species (ROS) levels can activate the mucolipin TRP channel 1 (TRPML1) in lysosomes and promote their exocytosis [35,36]. Increased oxidative stress induces lysosomal exocytosis instead of cell damage in T cells and astrocytes by elevating intracellular calcium levels [37,38]. In line with this, HIV-1 Tat promotes CATB release from astrocytes through lysosomal exocytosis, and it contributes to the astrocyte-mediated Tat neurotoxicity [39]. HIV-1 gp120 induces lysosomal exocytosis from human Schwann cells [40]. In U87MG glioblastoma cells, HIV-1 gp120 exposure increases ROS levels and promotes de-acidification and the positioning of lysosomes toward the plasma membrane [41]. Cinti and collaborators in 2017 demonstrated that in macrophages, HIV-1 infection promotes the movement of lysosomes to the cell periphery to facilitate HIV-1 budding and release and suggested that lysosomes fuse with the plasma membrane to assist in viral assembly at the inner plasma membrane [42]. In cancer, lysosomes accumulate at the cell periphery and secrete their contents, including CATB, to the extracellular milieu [43,44,45]. Moreover, an increased production of CATB and cathepsin L promotes lysosome positioning toward the cell periphery [45,46,47,48], and lysosomes at the cell periphery fuse with each other and then with the plasma membrane in a calcium-dependent manner [45,49]. However, whether HIV-1 infection of macrophages promotes CATB secretion through lysosomal exocytosis is unknown. 

Agonists of the CB2R have shown therapeutic potential against HIV-1 replication, neuroinflammation and neurotoxicity in several studies, which have been reviewed by different groups [50,51,52,53,54,55], and new supporting evidence continues to emerge [14,56]. In searching for strategies against CATB-induced neurotoxicity, we recently demonstrated that activating the CB2R with agonists decreases HIV-1 replication, CATB secretion, and neurotoxicity from HIV-infected macrophages [14]. CB2R agonists decrease iNOS and ROS generation in LPS-treated microglia by attenuating NF-κB activation in microglia [57]. The CB2R agonist, JWH-133, ameliorates the proinflammatory response in rats with induced lung injury by suppressing MAPKs and NF-κB signaling pathways [58]. In a mouse model of collagen-induced arthritis, JWH-133 polarized macrophages to an anti-inflammatory phenotype and inhibited RANKL-induced NF-κB activation in RAW264.7 macrophages [59]. This agonist also promotes the nuclear translocation of Nrf2 and increases the expression of HO-1 in macrophages following LPS treatment [60]. However, whether CB2R activation modulates NF-κB and Nrf2/ARE activation in HIV-infected macrophages is unknown. Additionally, the role of CB2R activation in lysosomal exocytosis is unknown. 

Therefore, we hypothesized that the HIV-1 infection of macrophages upregulates proteins associated with NF-κB signaling and lysosomal exocytosis, downregulates proteins associated with the Nrf2/ARE to promote oxidative stress, and both effects can be reduced by the CB2R agonist JWH-133. To test this hypothesis, we applied a quantitative proteomics approach using Tandem Mass Tag (TMT) and LC/MS/MS to HIV-infected MDM exposed to JWH-133 compared to controls, followed by Limma software (version 3.18) and Ingenuity Pathways Analyses (IPA). 

## 2. Results

### 2.1. JWH-133 Inhibits HIV-Induced Activation of Nrf2-Mediated Oxidative Stress Response and NF-κB Signaling

**Figure 1:**
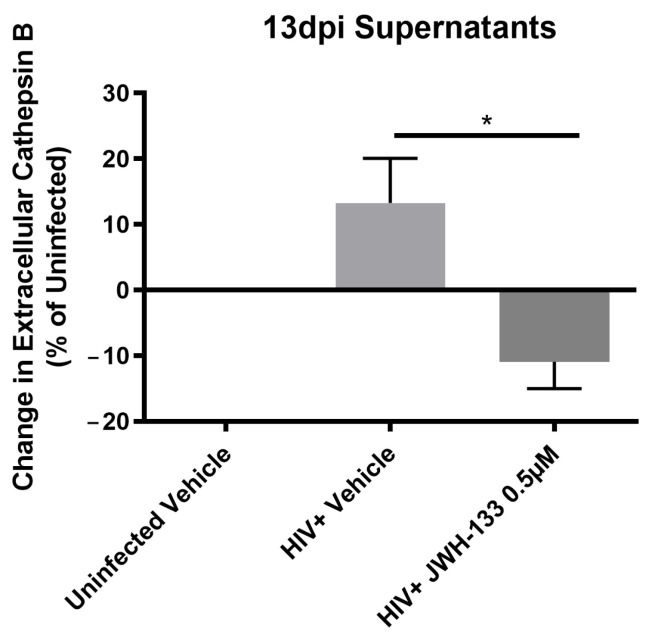
Total CATB levels in supernatants of MDM selected for TMT proteomics. MDM were cultured for 7 days and infected with HIV-1_ADA_. At day 1 post-infection (pi), MDM were treated with JWH-133 (0.5 μM) or vehicle control, and cultures were maintained until day 13 post-infection, replacing half of media with fresh media containing JWH-133 treatment every three days. Uninfected MDM treated with vehicle were used as controls. At day 13 pi, supernatants and lysates were collected. Total CATB levels were measured by ELISA. The percent change in total CATB levels compared to the uninfected vehicle-treated control per donor was calculated. The mean ± standard error of the mean (SEM) is shown. * *p* < 0.05.

In our previous studies, we have demonstrated that macrophages with increased CATB secretion after HIV infection are neurotoxic, whereas macrophages with decreased CATB secretion after HIV infection are unable to promote neurotoxicity [13]. In addition, we demonstrated that JWH-133 treatment decreased CATB levels in HIV-infected MDM [14]. Therefore, for this study, we selected a representative number of HIV-infected MDM cells (*n* = 3) with increased CATB secretion and treated with JWH-133 at 0.5 µM, lysed after 13 dpi, and stored at −80 °C (Figure 1). 

**Figure 2:**
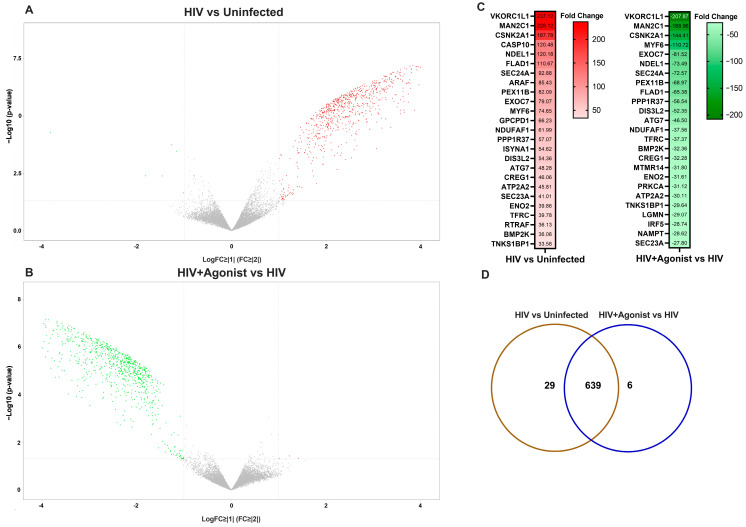
Volcano plots of differentially deregulated protein species. (**A**) Volcano plot depicting differentially abundant protein species for the HIV vs. Uninfected comparison. (**B**) Volcano plot depicting differentially abundant protein species for the HIV + Agonist vs. HIV comparison. Each upregulated protein specie is represented by a red symbol. Each downregulated protein specie is represented by a green symbol. Each protein specie that did not change is represented by a gray symbol. (**C**) Heatmap with the top significant differentially expressed proteins (*p*-value ≤ 0.05 and fold change ≥ |2|) between both groups ordered by the magnitude of the fold change. (**D**) Venn diagram showing the number of common and unique differentially abundant proteins for the two groups of comparisons with a fold change ≥ |2| and *p*-value ≤ 0.05 as identified by IPA’s comparison analysis.

Following bioinformatics analyses, we found that most differentially abundant proteins increased in HIV-infected macrophages when compared to uninfected cells as shown in volcano plots (Figure 2A). These proteins decreased in the presence of the HIV + Agonist, as represented in Figure 2B. A total of 1889 deregulated protein species were identified in the HIV-infected MDM compared to uninfected controls. Of these, 1821 were upregulated and 14 were downregulated, as shown in Table S1. When we compared the HIV + Agonist group to the HIV-infected group with no treatment, a total of 1821 protein species were deregulated, with eight protein species being upregulated with the agonist compared to 1813 being downregulated in absence of the agonist (Table S2). A heatmap was constructed with the top significant differentially expressed proteins (*p* value ≤ 0.05 and fold change ≥ |2|) between both groups ordered by the magnitude of the fold change (Figure 2C). After entering the data to IPA and performing group comparison analyses, we identified 639 proteins that were commonly deregulated by HIV infection and agonist treatment, whereas 29 proteins were deregulated by HIV only and 6 by JWH-133 treatment (Figure 2D). In addition, JWH-133 reversed the effect (upregulated) of the following proteins whose expression was downregulated in HIV-infected untreated MDM: sulfotransferase (accession ID: E9PKR8 and H3BQX5), desmin (accession ID: P17661), phosphopyruvate hydratase (accession ID: E5RGZ4), and beta-enolase (accession ID: P13929) (Tables S1 and S2).

**Figure 3:**
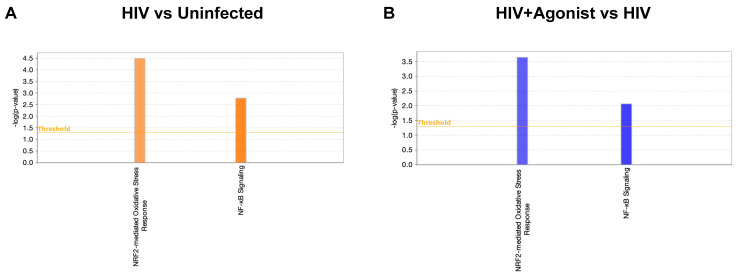
Deregulated canonical pathways. (**A**) Canonical pathways of Nrf2-mediated oxidative stress response and NF-κB signaling for the comparison of HIV vs. Uninfected. (**B**) Canonical pathways of Nrf2-mediated oxidative stress response and NF-κB signaling for the comparison of HIV + Agonist vs. HIV. Orange color represents a predicted activated pathway (positive z-score). Blue color represents a predicted inhibited pathway (negative z-score). This figure was obtained from IPA.

Using IPA, we searched for canonical pathways associated with Nrf2 and NF-κB signaling and found that HIV infection of macrophages activated the mechanisms of the Nrf2-mediated oxidative stress response and NF-κB signaling pathways as predicted by IPA (Figure 3A). All these mechanisms were inhibited by JWH-133 treatment (Figure 3B). The 16 commonly deregulated proteins between the two comparisons associated with Nrf2-mediated oxidative stress response are shown in Table 1. Illustrations of this canonical pathway depicting the deregulated proteins and their cellular locations are shown for the comparison of HIV+ vs. Uninfected group (Figure S1) and for the comparison of HIV + Agonist vs. Untreated HIV-infected group (Figure S2). Most proteins were upregulated by HIV infection and downregulated by JWH-133 CB2R agonist treatment. Among the deregulated proteins, the top five proteins with the highest fold change expression values were the following: protein kinase C alpha (*PRKCA*), 3-hydroxyacyl-CoA-dehydratase 3 (*HACD3*), cullin 3 (*CUL3*), phosphoinositide-3-kinase regulatory subunit 2 (*PIK3R2*), and DnaJ heat shock protein family (Hsp40) member C7 (*DNAJC7*). PRKCA phosphorylates Nrf2 in macrophages [61]. HACD3 is an enzyme, which is involved in lipid metabolism that is expressed during Nrf2 activation [62]. *CUL3*, with the help of the Roc1 complex, binds keap1 and ubiquitinates Nrf2, leading to its degradation under a resting state [63]. PI3K, of which PIK3R2 is a subunit, modifies actin in the cytoplasm, allowing its binding to Nrf2 and translocation into the nucleus during oxidative stress [64]. The PI3K/Akt activation also leads to Nrf2 activation under oxidative stress [65]. The product of *DNAJC7* is a heat-shock protein (Hsp40) that is expressed during Nrf2 activation [62].

The 12 commonly deregulated proteins between the two comparison groups (HIV Agonist treated vs. HIV untreated) that were associated with NF-κB signaling are shown in Table 2. Illustrations of this canonical pathway depicting the deregulated proteins and their cellular locations are shown for the HIV+ vs. Uninfected group comparison (Figure S3) and for the HIV + Agonist vs. HIV comparison (Figure S4). These proteins were upregulated by HIV and downregulated by the CB2R agonist, JWH-133. Among the deregulated proteins, the top five proteins with the highest fold change expression values were the following: casein kinase 2 alpha 1 (*CSNK2A1*), A-Raf proto-oncogene serine/threonine kinase (*ARAF*), mitogen-activated protein kinase kinase kinase kinase 4 (*MAP4K4*), phosphoinositide-3-kinase regulatory subunit 2 (*PIK3R2*), and tyrosyl-DNA phosphodiesterase 2 (*TDP2*). CSNK2A1, during TNF-α and IL-1 receptors activation, phosphorylates the p65 (RelA) NF-κB subunit in the cytoplasm, leading to its translocation to the nucleus [66,67,68,69,70]. A-Raf activates MEKK1 in the cytoplasm, leading to NF-κB activation [71]. MAP4K4, also known as NIK, activates the IKK-α [72]. PIK3R2 is part of the PI3K complex that activates AKT proteins [73]. TDP2 binds to TNF-α receptor associated proteins to inhibit NF-κB activation through TRAF6; however, it does not inhibit IKK-α and the p65-mediated activation of NF-κB [74].

### 2.2. JWH-133 Decreases the Expression of Proteins Associated with Lysosomal Exocytosis

**Figure 4:**
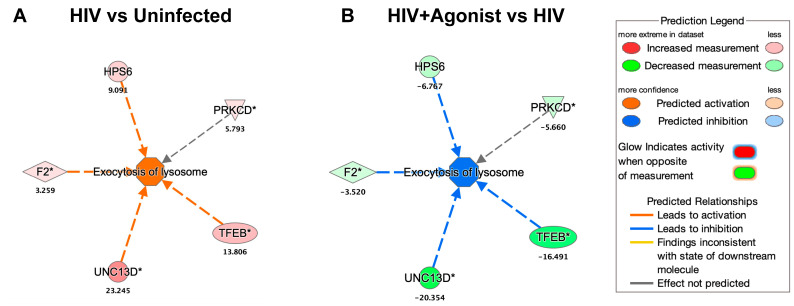
Node of proteins associated with lysosomal exocytosis. (**A**) Deregulated proteins associated with the function of “Exocytosis of Lysosome” for the HIV vs. Uninfected comparison. (**B**) Deregulated proteins associated with the function of Exocytosis of Lysosome for the HIV + Agonist vs. HIV comparison. The expression fold change value is shown below each protein’s gene symbol. A solid line indicates a direct interaction, and a dashed line indicates an indirect interaction. An asterisk indicates that multiple identifiers in the dataset file map to a single gene in the Global Molecular Network. Transcription factor EB (*TFEB*), coagulation factor II (*F2*), protein unc-13 homolog D (*UNC13D*), Hermansky–Pudlak syndrome 6 protein (*HPS6*), and protein kinase C delta (*PRKCD*). This figure was obtained from IPA.

By searching for the deregulated proteins associated with the function of “Exocytosis of Lysosome” in IPA, we found that HIV infection and JWH-133 commonly deregulated the expression of five proteins (Figure 4). These proteins were upregulated by HIV and downregulated by JWH-133. The deregulated proteins were transcription factor EB (*TFEB*), coagulation factor II (F2), protein unc-13 homolog D (*UNC13D*), Hermansky–Pudlak syndrome 6 protein (*HPS6*), and protein kinase C delta (*PRKCD*). TFEB is the master regulator of lysosomal biogenesis, and its activation promotes lysosomal exocytosis [75]. CATB participates in TFEB-mediated lysosomal exocytosis in monocytes exposed to high glucose [76]. UNC13D participates in the LPS-induced lysosomal exocytosis in neutrophils [77]. Alpha thrombin, a product of F2, increases the secretion of lysosomes in platelets [78]. HPS6 participates in lysosomal exocytosis from platelets [78]. PRKCD activation promotes lysosomal exocytosis in cytotoxic CD8^+^ T cells [79].

### 2.3. JWH-133 Decreases the Expression of CATB and Associated Proteins

**Figure 5:**
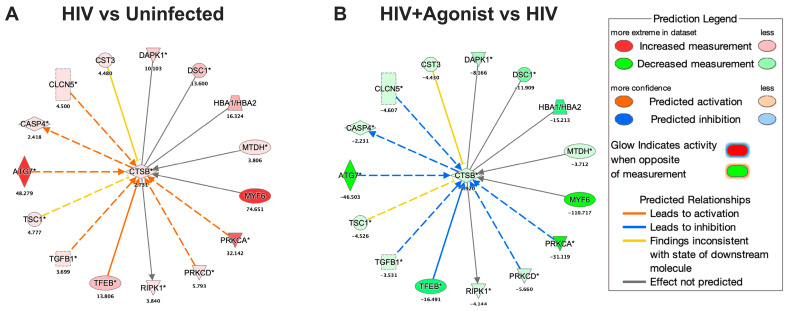
Node of proteins associated with CATB. (**A**) Deregulated proteins associated with CATB for the HIV vs. Uninfected comparison. (**B**) Deregulated proteins associated with CATB for the HIV + Agonist vs. HIV comparison. The expression fold change value is shown below each protein’s gene symbol. A solid line indicates a direct interaction, and a dashed line indicates an indirect interaction. An asterisk indicates that multiple identifiers in the dataset file map to a single gene in the Global Molecular Network. Transforming growth factor beta 1 (*TGFβ1*), TSC complex subunit 1 (*TSC1*), autophagy-related 7 (*ATG7*), caspase 4 (*CASP4*), chloride voltage-gated channel 5, cystatin C (*CST3*), death-associated protein kinase 1 (*DAPK1*), desmocollin 1 (*DSC1*), hemoglobin subunit alpha 1/2 (*HBA1*/*HBA2*), metadherin (*MTDH*), myogenic factor 6 (*MYF6*), and receptor interaction serine/threonine kinase 1 (*RIPK1*). *CTSB* (gene symbol) was used to abbreviate CATB in this figure. This figure was obtained from IPA.

HIV infection of macrophages increased the intracellular expression of CATB by 2.7-fold, whereas JWH-133 decreased its expression by −2.9-fold (Figure 5). We searched for the deregulated proteins associated with CATB in IPA and found that HIV infection and JWH-133 commonly deregulated the expression of 15 proteins. These proteins were upregulated by HIV and downregulated by JWH-133. The deregulated proteins were the following: *TFEB*, transforming growth factor beta 1 (*TGFβ1*), TSC complex subunit 1 (*TSC1*), autophagy-related 7 (*ATG7*), caspase 4 (*CASP4*), chloride voltage-gated channel 5, cystatin C (*CST3*), death-associated protein kinase 1 (*DAPK1*), desmocollin 1 (*DSC1*), hemoglobin subunit alpha 1/2 (*HBA1/HBA2*), metadherin (*MTDH*), myogenic factor 6 (*MYF6*), *PRKCA*, *PRKCD*, and receptor interaction serine/threonine kinase 1 (*RIPK1*). The top 5 proteins with the highest expression fold changes were the following: *MYF6*, *ATG7*, *PRKCA*, *HBA1/HBA2*, and *TFEB*. In mice, MYF6 regulates CATB mRNA expression [80]. CATB participates in ATG7-induced nod-like receptor 3 (NLRP3)-inflammasome activation in a rat insulinoma cell line [81]. PRKCA mediates the expression of CATB in a human breast cancer cell line [82]. In mice, CATB interacts with HBA1 [83]. TFEB increases the protein expression of CATB in Hela cells [84].

## 3. Discussion

In the past, we have shown that the expression of CATB, a proinflammatory lysosomal enzyme, increases in post-mortem brain tissues, plasma, and monocytes of individuals with HAND [7,8]. The increased secretion of this enzyme from in vitro HIV-infected macrophages leads to neuronal apoptosis [7,9,10,11,12,13]. Our studies have also shown that CATB neurotoxicity is decreased by the addition of an inhibitor or antibody against CATB [7,9,10,11,12,13,14,85]. In addition, we have recently found that CB2R agonists can decrease the secretion and neurotoxic potential of CATB from HIV-infected MDM [14]. However, the mechanisms by which HIV-1 infection and CB2R activation modulate CATB secretion in macrophages remained unknown. Using a quantitative proteomics approach, we have uncovered proteins and pathways related to the expression and secretion of CATB from MDM, such as NF-κB signaling, Nrf2-oxidative stress response, and lysosomal exocytosis pathways. We hypothesized that HIV-1 activates an antioxidant response through Nrf2 activation at a relatively later stage during their infection to counteract the damaging effects of oxidative stress and ensure the macrophage reservoir survival. In HIV-1 transgenic mice, an elevation of mRNA expression of antioxidant enzymes such as heme oxygenase-1 and glutathione s-transferase was observed despite the downregulation of Nrf2 mRNA expression by 50% of its normal levels [86]. The authors suggested that even at these lower expression levels, Nrf2 was sufficiently active to mount an antioxidant response for protection against oxidative stress. Our study is consistent with their observations, as we observed that HIV-1 infection elevated the expression of heme oxygenase-1 (Table 1). In addition, a recent study demonstrated that Nrf2 activation inhibits apoptosis in HIV-infected macrophages [87]. In contrast, other studies have reported a decreased expression of heme oxygenase-1 in HIV-infected macrophages and in certain regions of the brain of patients with HAND [26,88,89]. However, they also reported an elevation of Nrf2 expression in in vitro HIV-infected macrophages and suggested that in the dorsolateral prefrontal cortex of patients with HAND, the Nrf2/ARE activity is not affected by HIV-1 infection [88,89]. These results suggest that the relation of HIV-1 infection with the Nrf2/ARE is complex and might be context-dependent. On the other hand, CB2R activation by JWH-133 might have prevented Nrf2 activation by inhibiting HIV-1 RT activity, a known inducer of Nrf2, and by maintaining control of HIV-1 replication and oxidative stress [31]. This hypothesis is supported by a previous study that demonstrated that JWH-133 inhibits HIV-1 RT activity in primary HIV-infected macrophages [90].

HIV-infected macrophages activated NF-κB signaling, and this effect was decreased by JWH-133. Thus, these results align with previous studies showing that the activation of NF-κB in HIV-infected macrophages promotes the viral reservoir persistence [91,92] as well as with studies demonstrating that CB2R activation attenuates NF-κB activation in macrophages, leading to a decreased oxidative stress and neuroprotection [93,94]. Since NF-κB activates the HIV-1 long-terminal repeat (LTR), the inhibition of NF-κB signaling by JWH-133 might also contribute to the downregulation of HIV-1 replication as demonstrated in previous studies, including ours [14,90,95]. Furthermore, HIV-1 infection increased the expression of proteins associated with the lysosomal exocytosis mechanism, whereas JWH-133 decreased the expression of all these proteins. These results are consistent with previous studies in astrocytes where exposure to HIV-1 Tat promoted the release of CATB and neurotoxicity through lysosomal exocytosis [39]. However, this is the first study suggesting that HIV-1 infection in macrophages promotes lysosomal exocytosis and that it is attenuated by JWH-133 treatment, which is a significant advance for future treatment modalities. In a previous TMT-based proteomics study, we observed an increased CATB expression in macrophages from HIV-seropositive women with impaired cognition compared to asymptomatically impaired HIV-seropositive woman after 7 days in culture, suggesting that CATB protein expression is linked to cognitive impairment in HIV-seropositive women [96]. In this study, we found that intracellular CATB levels were increased by HIV-1 infection and decreased by JWH-133 treatment. These results differ from previous in vitro studies in our laboratory, which demonstrated that intracellular CATB protein levels were unaffected in HIV-infected macrophages at day 12 pi compared to healthy MDM controls as determined by Western blot, even when its mRNA expression and extracellular secretion levels were increased [7]. However, since we used 13pi lysates for these studies, it is possible that there are time-dependent differences in CATB intracellular expression. Our results suggest that JWH-133 decreases CATB secretion, in part, by downregulating its intracellular expression. Additionally, we found proteins associated with CATB that were increased by HIV-1 infection and decreased by JWH-133. One limitation of this study is that the significant proteins affected by JWH-133 could not be validated by other methods such as Western blot or qPCR. This study focused on the pathways affected by the agonist, since our experiments were limited by the amount of MDM protein lysates to perform further validations. This could be subject to future studies. In this study, we did not consider the analyses of uninfected MDM treated with JWH-133, as uninfected MDM do not induce neurotoxicity.

**Figure 6:**
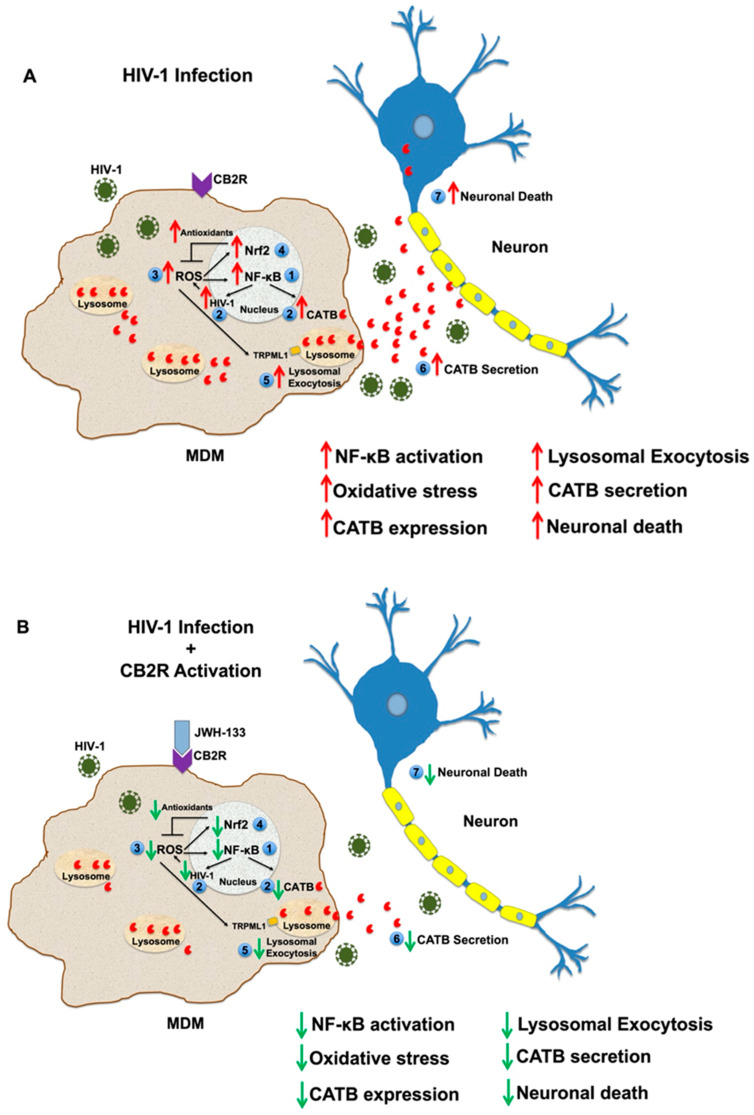
Proposed mechanism of JWH-133 in CATB secretion and neurotoxicity from HIV-infected MDM. (**A**) By day 13 pi, HIV-1 infection induces the activation of NF-κB signaling (1). NF-κB activation increases CATB expression and HIV-1 replication (2), which allows the generation of ROS and maintains NF-κB activation (3). Increased oxidative stress promotes the activation of the Nrf2-mediated antioxidant response, which reduces ROS to moderate levels, ensuring the survival of the macrophage viral reservoir (4). Moderate levels of ROS activate TRPML1, inducing lysosomal exocytosis (5) and the release of CATB to the extracellular milieu (6). Increased secretion of CATB leads to neuronal death (7). (**B**) Continuous treatment of HIV-infected MDM with CB2R agonist JWH-133 since day 1 pi attenuates the activation of NF-κB (1), reducing CATB expression and HIV-1 replication (2). Therefore, it decreases the generation of ROS (3), the Nrf2-mediated antioxidant response (4), lysosomal exocytosis (5), CATB secretion (6), and neuronal death (7). Red arrows indicate upregulation. Green arrows indicate downregulation.

In summary, the activation of NF-κB, oxidative stress, and lysosomal exocytosis might be involved in the mechanism of CATB secretion and neurotoxicity from HIV-infected macrophages. We propose that HIV-1 infection induces NF-κB activation, which drives the expression of CATB and HIV-1 replication (Figure 6). HIV-1 replication increases ROS and activates the Nrf2-mediated antioxidant response to counteract the damaging effects of oxidative stress and ensure the reservoir survival. Moderate levels of ROS drive the exocytosis of lysosomes by activating the TRPML1 calcium channel in the lysosomes, promoting increased secretion of CATB and neurotoxicity. The hypothesis that moderate levels of oxidative stress promote lysosomal exocytosis from HIV-infected MDM is supported by a previous study [35]. This study demonstrated that high levels of ROS induced by HIV-1 infection inhibit lysosomal exocytosis, promoting cell damage, whereas moderate levels of ROS induced lysosomal exocytosis as a mechanism of cell repair by activating TRPML1 [35]. Therefore, these mechanisms represent potential targets for treatment against HIV-induced neurotoxicity. Most importantly, CB2R activation represents a potential therapeutic target against HAND.

## 4. Materials and Methods

### 4.1. Isolation of Macrophages, HIV-1_ADA_ Infection, and Treatments with JWH-133 Agonist

Peripheral blood mononuclear cells (PBMCs) were isolated from healthy women donors over 21 years of age. The study was managed under the approval of the University of Puerto Rico, Medical Sciences Campus Institutional Review Board (IRB) (Protocol #0720116). Signed informed consents were obtained from each donor by the Code of Ethics of the World Medical Association and the institutional guidelines and regulations. PBMCs were cultured in T25 flasks at a concentration of 10 × 10^6^ cells/flask, and (MDM) were isolated by adherence after seven days in fetal bovine serum 10% (FBS), human serum 1%, RPMI, and 100 U/mL pen/strep (Sigma, St. Louis, MO, USA). The media were changed every three days. On day 7 of culture, HIV-1_ADA_ was added to the T25 flasks at an MOI of 0.1, and MDM were incubated overnight at 37 °C in 5% CO_2_. Thereafter, cells were washed with media and treated with CB2R agonist JWH-133 at 0.5 µM, exchanging half of the media with fresh media containing JWH-133 (0.5 µM) every three days on days 3, 6, and 9 pi. At day 12 pi, all the media were replaced with serum-free media, and cells were incubated at 37 °C in 5% CO_2_ for 24 h. At day 13 pi, this supernatant was collected and saved at −80 °C for future neurotoxicity experiments, which require serum-free conditions. In parallel, at day 13 pi, MDM lysates were collected and saved at −80 °C for TMT labeling proteomics. The rationale for using day 13 pi as our endpoint is because previous studies in our laboratory have demonstrated that CATB is neurotoxic after twelve days post-infection and up to day 13 pi, which is the latest time-point at which we tested its neurotoxic potential [7,9,10,11,12,13,14].

### 4.2. Total CATB ELISA

Total CATB levels were measured in MDM supernatants from 13 dpi using ELISA according to the manufacturer’s instructions (R&D systems, Minneapolis, MN, USA, respectively).

### 4.3. Preparation of MDM Lysates for TMT Labeling

MDM lysates from three donors (*n* = 3) showing increased CATB levels after HIV infection at day 13 post-infection were selected for Tandem Mass Tag (TMT) labeling, following an already developed protocol at the UPR-MSC Translational Proteomics Center [97]. In summary, MDM were washed twice with phosphate-buffered saline (PBS) and incubated with 100 µL of lysis buffer (5 mM Tris-HCl at pH 8.0, 0.1% Triton X-100), which contained 5 µL of protease inhibitor cocktail (AEBSF, Aprotinin, Bestatin, ethylenediaminetetraacetic acid (EDTA), and leupeptin). Cells were incubated for 30 min on ice and scraped for protein extraction. After that, cells were vortexed and centrifuged at 4 °C for 10 min at 1500 rpm. Finally, the supernatants containing the intracellular proteins were collected and saved at −80 °C.

For total protein quantitation of MDM lysates, we performed the bicinchoninic acid (BCA) test (DC protein assay, Bio-Rad, Hercules, CA, USA) following the manufacturer’s instructions (Bio-Rad, La Jolla, CA, USA). Samples were read at 450 nm in the Varioskan Flash Spectral Reader (Thermo Fisher Scientific, Bannockburn, IL, USA). Twenty micrograms of protein were used for TMT analysis. Three donors and three conditions (HIV-uninfected with vehicle, HIV-infected with vehicle, and HIV-infected with agonist) for a total of 9 samples were used for TMT10-plex.

In a sample tube with approximately 20 µg of mixed cell lysate protein, 50 µL of 10% sodium dodecyl sulfate (SDS) was added to initiate the acetone precipitation. Then, it was heated for 15 min at 70 °C, and cold acetone (~1 mL) was added to the sample until a final dilution of 15% was achieved. Samples were incubated overnight at −20 °C. The next day, samples were centrifuged at 10,000× *g* for 10 min, the supernatants were removed, and a sample buffer containing 2× Laemmli buffer + β-mercaptoethanol (Bio-Rad, USA) was added. Proteins were rehydrated with sample buffer and heated for 10 min at 70 °C. In a pre-made gel (Mini-PROTEAN TGX 4–20%), the samples were run at 200 volts for 10 min. Gels were stained using Biosafe Coomassie G-250 staining and documented using Chemi-Doc XRS+ (Bio-Rad, La Jolla, CA, USA). Gels images are presented in Figure S5. Coomassie-stained proteome bands were cut manually in 1 mm^3^ cubes. For destaining, a mixture of 50% acetonitrile and 50 mM ammonium bicarbonate was added; then, proteins were reduced using 25 mM dithiothreitol (DTT) in 50 mM ammonium bicarbonate for 30 min at 55 °C and alkylated with 10 mM iodoacetamide (IAA) in 50 mM ammonium bicarbonate for 45 min in the dark. Samples were digested with sequencing grade-modified trypsin (Promega, Madison, WI, USA) in 50 mM ammonium bicarbonate at a ratio of 1:50 (trypsin/protein) overnight at a temperature of 37 °C for a maximum of 16 h. The peptides were extracted from the gel pieces using 150 µL of a mixture of 50% acetonitrile and 2.5% formic acid in water and 150 µL of 100% acetonitrile. Digests were dried for subsequent TMT labeling.

### 4.4. TMT Labeling

TMT labeling was performed following the manufacturer’s instructions (Thermo Fisher Scientific) and according to Borges-Vélez et al., 2021 [97]. One TMT10-plex platform was required, accommodating three donors with their conditions in each platform with the addition of a pool composed of peptides from uninfected vehicle controls in the same label across platforms. The specific TMT tags used for each sample are described in Table S3. Triethylammonium bicarbonate (TEAB) was used to reconstitute the dried peptides in 100 mM buffer and then labeled with the TMT10-plex reagents. The TMT reagents were resuspended in 41 µL of anhydrous acetonitrile (99.9%), added to each respective sample, and incubated for one hour at room temperature. To quench the reaction, 5% hydroxylamine was added and incubated for 15 min. Finally, equal amounts of labeled sample were mixed to generate a final pool that was dried and subjected to fractionation.

The fractionation was performed using the Pierce High pH Reversed-Phase Peptide Fractionation Kit (Thermo Fisher Scientific) and following manufacturer’s instructions. Briefly, the column was conditioned twice using 300 μL of acetonitrile, centrifuged at 5000× *g* for 2 min, and the steps were repeated using 0.1% trifluoroacetic acid (TFA). Each TMT labeled pool was reconstituted in 300 μL of 0.1% TFA, loaded onto the column and washed with water and 5% acetonitrile/0.1% triethylamine (TEA). Thereafter, the sample was eluted 8 times into 8 different vials using a series of elution solutions with different acetonitrile/0.1% TEA percentages as suggested by the manufacturer. Fractions were dried and stored for mass spectrometric analysis.

### 4.5. Mass Spectrometry Analyses

Mass spectrometry analyses were performed according to Borges-Vélez et al., 2021 [97]. An HPLC system (Easy nLC 1200) (Thermo Fisher Scientific) was used for peptides separation. First, the peptides are loaded onto a Pico Chip H354 REPROSIL-Pur C18-AQ 3 µM 120 A (75 µm × 105 mm) chromatographic column (New Objective, Littleton, MA, USA). In a total gradient time of 128 min, separation was obtained at a rate of 300 nL/min as follows: 7–25% 0.1% formic acid in 80% acetonitrile (Buffer B) for 102 min, 25–60% Buffer B for 20 min and 60–95% for 6 min. After separating the peptides, they were sprayed and analyzed using a mass spectrometer operated in positive polarity mode and data-dependent mode using a Q-Exactive Plus (Thermo Fisher Scientific, Bannockburn, IL, USA). The MS1 (full scan) was measured in a range of 375 to 1400 *m*/*z* with a resolution of 70,000. To select the ten most intense ions for HCD fragmentation and MS2 (MS/MS) analysis with a resolution of 35,000, a dynamic exclusion parameter was created in 30.0 s with a repeat count of three.

### 4.6. Protein Identification and Quantitative Analyses

The protein identification process has been made following the protocol established by the Translational Proteomics Center [97]. MS/MS raw data files were searched using Proteome Discoverer version 2.5 (Thermo Fisher Scientific) with a SEQUEST HT algorithm, and proteins were identified using the human protein database from the same software’s protein knowledgebase. Trypsin was included in the search as an enzyme for proteolysis with a range of 6–144 in peptide length. The precursor mass tolerance was set at 20 ppm and 0.02 Da for the fragment mass tolerance. Oxidation +15.995 Da (M) was included as a dynamic modification. The static modifications included were carbamidomethyl +57.021 Da (C), TMT reagents +229.163 Da (Any N-terminal end, K). The false discovery rate was set at 0.01 (strict) and 0.05 (relaxed). The raw protein files obtained were exported from the Proteome Discoverer software to .xls format using Microsoft Excel Program 2016 (Los Angeles, CA, USA) for further bioinformatic analyses [62].

### 4.7. Bioinformatics and Statistical Analyses

Following an already developed protocol at the Translational Proteomics Center [97], we used the Bioconductor R-Limma (version 3.18) package to carry out statistical analyses of protein abundances [98]. In summary, two different comparisons (experimental case/control) of protein abundances were analyzed with the Limma software (version 3.18) to calculate their respective fold changes and *p*-values. Single-channel analyses included abundances between the following: HIV+/Vehicle vs. Uninfected/Vehicle, and HIV+/Agonist vs. HIV+/Vehicle. Statistically significant differentially expressed proteins met the following parameters: fold-change (FC) values greater than or equal to |2| (i.e., FC  ≥ |2|) and a *p*-value less than or equal to 0.05 (i.e., *p*-value ≤ 0.05, 95% confidence). Significantly differentially expressed proteins were entered into IPA (QIAGEN, Inc., Germantown, MD, USA) to perform enrichment analyses based on the canonical pathways “NF-κB signaling” and “Nrf2-mediated oxidative stress response”, and the nodes “Exocytosis of lysosome”, and CATB (“CSTB”). In addition, the molecule activity predictor (MAP) was included in the analyses.

## 5. Conclusions

This is the first study that demonstrates that a CB2R agonist downregulates the expression of proteins associated with NF-κB signaling, Nrf2-mediated oxidative stress response, and lysosomal exocytosis in HIV-infected macrophages, indicating that these proteins are involved in the secretion of CATB. As proposed in Figure 6, the use of JWH-133 warrants future in vivo studies to test its potential as a therapeutic ligand against HIV-induced neurotoxicity and HAND. The results obtained in this study provide a foundation for future studies on the mechanisms driving HIV-induced neurotoxicity from macrophages. This study reveals alternative strategies for treatment against HIV-induced neurotoxicity and HAND that should be validated in human studies.

## Figures and Tables

**Table 1:** Deregulated proteins in Nrf2-mediated oxidative stress response canonical pathway. Commonly deregulated proteins associated with Nrf2-mediated oxidative stress response canonical pathway between both groups of comparisons. Red color indicates upregulated proteins, while green color downregulated proteins. Expr = Expression.

		HIV vs. Uninfected		HIV + Agonist vs. HIV	
Symbol	Entrez Gene Name	Expr Fold Change	Expr *p*-Value	Expr Fold Change	Expr *p*-Value
*PRKCA*	protein kinase C alpha	32.142	4.63 × 10^−8^	−31.119	3.94 × 10^−6^
*HACD3*	3-hydroxyacyl-CoA dehydratase 3	25.127	1.51 × 10^−7^	−23.856	1.07 × 10^−7^
*CUL3*	cullin 3	9.871	6.53 × 10^−7^	−9.554	4.99 × 10^−6^
*PIK3R2*	phosphoinositide-3-kinase regulatory subunit 2	9.386	7.64 × 10^−7^	−9.018	5.89 × 10^−6^
*DNAJC7*	DnaJ heat shock protein family (Hsp40) member C7	7.967	1.12 × 10^−6^	−8.220	1.40 × 10^−5^
*DNAJC11*	DnaJ heat shock protein family (Hsp40) member C11	6.470	1.67 × 10^−5^	−6.012	1.01 × 10^−6^
*PRKCD*	protein kinase C delta	5.793	1.29 × 10^−6^	−5.660	5.75 × 10^−6^
*HMOX1*	heme oxygenase 1	5.773	2.78 × 10^−6^	−5.579	2.41 × 10^−6^
*STIP1*	stress induced phosphoprotein 1	5.593	4.72 × 10^−6^	−5.338	2.06 × 10^−6^
*FTH1*	ferritin heavy chain 1	4.999	2.25 × 10^−6^	−5.082	1.07 × 10^−4^
*ACTG2*	actin gamma 2, smooth muscle	4.746	1.42 × 10^−5^	−4.700	4.70 × 10^−5^
*DNAJC8*	DnaJ heat shock protein family (Hsp40) member C8	4.268	1.81 × 10^−5^	−4.202	1.20 × 10^−5^
*DNAJB12*	DnaJ heat shock protein family (Hsp40) member B12	4.025	9.19 × 10^−5^	−3.563	8.55 × 10^−6^
*PIK3CB*	phosphatidylinositol-4,5-bisphosphate 3-kinase catalytic subunit beta	3.723	5.97 × 10^−6^	−4.084	6.96 × 10^−6^
*SQSTM1*	sequestosome 1	3.639	6.16 × 10^−5^	−3.879	6.59 × 10^−6^
*GCLC*	glutamate-cysteine ligase catalytic subunit	3.280	2.52 × 10^−3^	−3.711	3.33 × 10^−4^

**Table 2:** Deregulated proteins in NF-κB signaling canonical pathway. Commonly deregulated proteins associated with NF-κB signaling canonical pathway between both groups of comparisons. Red color indicates upregulated proteins while green color indicates downregulated proteins. Expr = Expression.

		HIV vs. Uninfected		HIV + Agonist vs. HIV	
Symbol	Entrez Gene Name	Expr Fold Change	Expr *p*-Value	Expr Fold Change	Expr *p*-Value
*CSNK2A1*	casein kinase 2 alpha 1	187.779	1.01 × 10^−8^	−144.412	1.16 × 10^−7^
*ARAF*	A-Raf proto-oncogene, serine/threonine kinase	85.425	2.27 × 10^−8^	−13.087	1.90 × 10^−5^
*MAP4K4*	mitogen-activated protein kinase kinase kinase kinase 4	22.938	2.13 × 10^−7^	−15.934	1.12 × 10^−6^
*PIK3R2*	phosphoinositide-3-kinase regulatory subunit 2	9.386	7.64 × 10^−7^	−9.018	5.89 × 10^−6^
*TDP2*	tyrosyl-DNA phosphodiesterase 2	6.952	3.23 × 10^−6^	−7.308	9.59 × 10^−6^
*EIF2AK2*	eukaryotic translation initiation factor 2 alpha kinase 2	6.464	8.41 × 10^−6^	−6.394	6.53 × 10^−6^
*TRADD*	TNFRSF1A associated via death domain	6.013	1.54 × 10^−6^	−4.772	8.51 × 10^−6^
*IGF2R*	insulin like growth factor 2 receptor	5.983	3.18 × 10^−6^	−5.753	3.25 × 10^−6^
*PRKACA*	protein kinase cAMP-activated catalytic subunit alpha	4.901	2.52 × 10^−6^	−4.533	2.30 × 10^−6^
*RIPK1*	receptor interacting serine/threonine kinase 1	3.84	9.41 × 10^−6^	−4.144	3.88 × 10^−6^
*PIK3CB*	phosphatidylinositol-4,5-bisphosphate 3-kinase catalytic subunit beta	3.723	5.97 × 10^−6^	−4.084	6.96 × 10^−6^
*MYD88*	MYD88 innate immune signal transduction adaptor	2.785	1.36 × 10^−4^	−2.638	1.20 × 10^−2^

## Data Availability

Most of the data generated or analyzed during this study are included in this published article. All the proteomics raw datasets generated in the current study have been deposited in the ProteomeXchange [99]. Consortium via the PRIDE [100], a partner repository with a dataset identifier: Project accession: PXD048464; Project DOI: 10.6019/PXD04.8464.

## Supplementary Materials

The supporting information can be downloaded at https://www.mdpi.com/article/10.3390/ijms25063246/s1.
