# Supplementary material for: Quantitative Proteomics Reveal That CB2R Agonist JWH-133 Downregulates NF-κB Activation, Oxidative Stress, and Lysosomal Exocytosis from HIV-Infected Macrophages
Source: Int J Mol Sci. 2024 Mar 13;25(6):3246. doi: 10.3390/ijms25063246 (PMC10970132; doi:10.3390/ijms25063246)
Supplement: Supplementary file 1 [file ijms-25-03246-s001.zip › Supplementary Figures S1-S5.pdf]

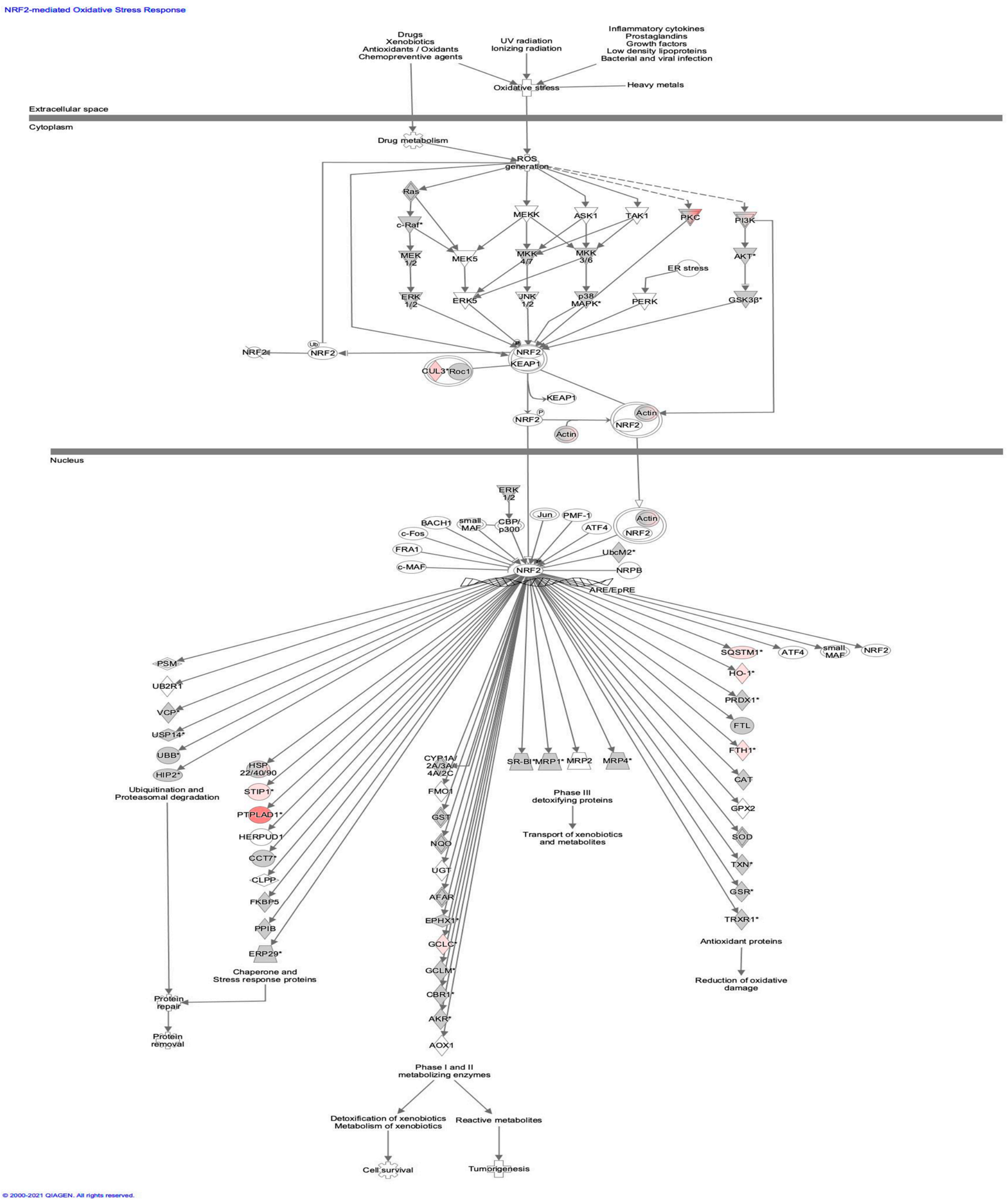

Figure S1. Canonical Pathway of Nrf2-mediated Oxidative Stress Response of the HIV infected group compared to uninfected controls. Red symbols represent upregulated proteins. Gray symbols represent unregulated proteins in the dataset. White symbols represent proteins absent in the dataset. Higher color intensity = higher absolute value of fold change expression. This figure was obtained from IPA.

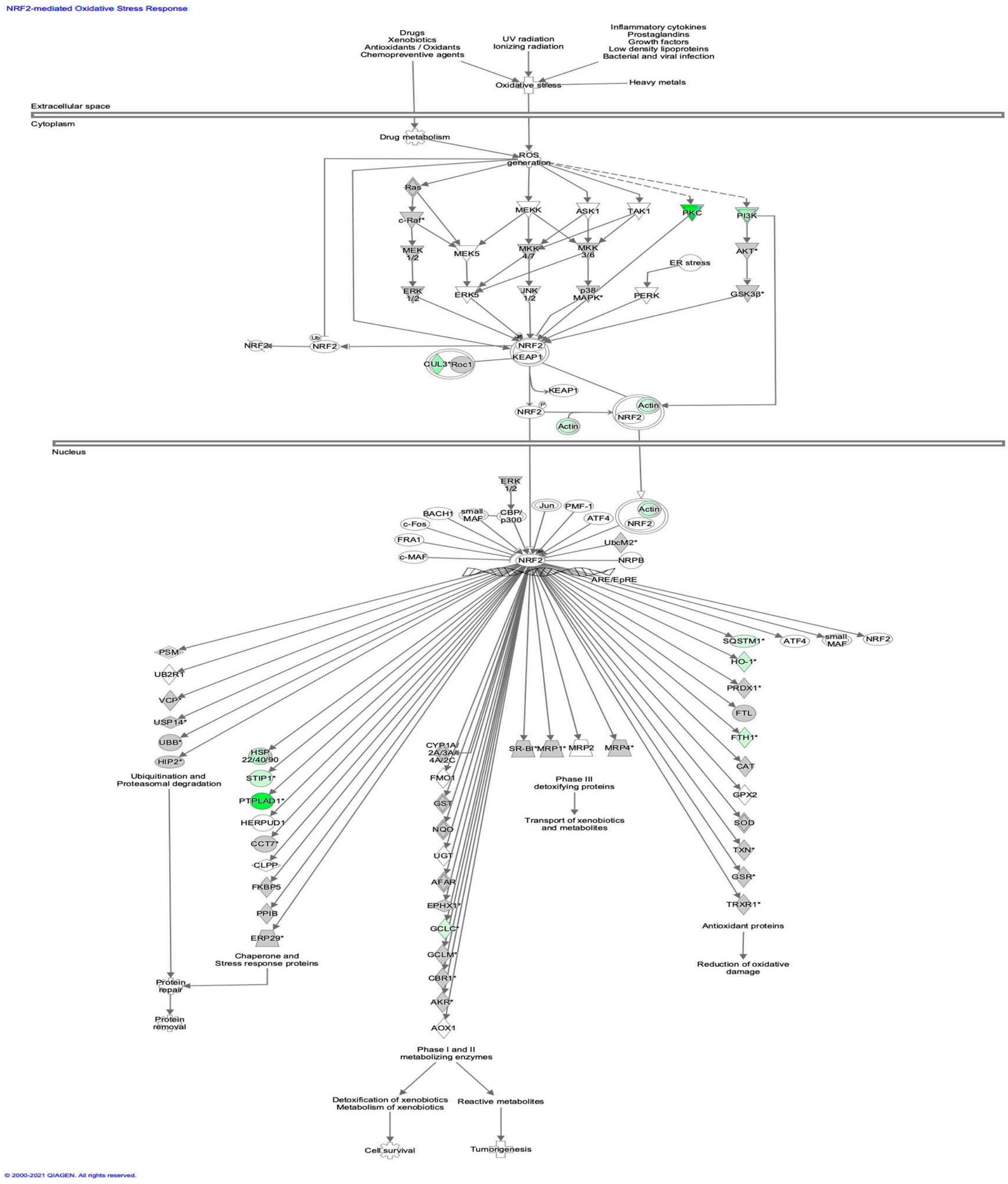

Figure S2. Canonical pathway of Nrf2-mediated oxidative stress response for the HIV + agonist group compared to untreated HIV. Green symbols represent downregulated proteins. Gray symbols represent unregulated proteins in the dataset. White symbols represent proteins absent in the dataset. Higher color intensity = higher absolute value of fold change expression. This figure was obtained from IPA.

NF-κB Signaling

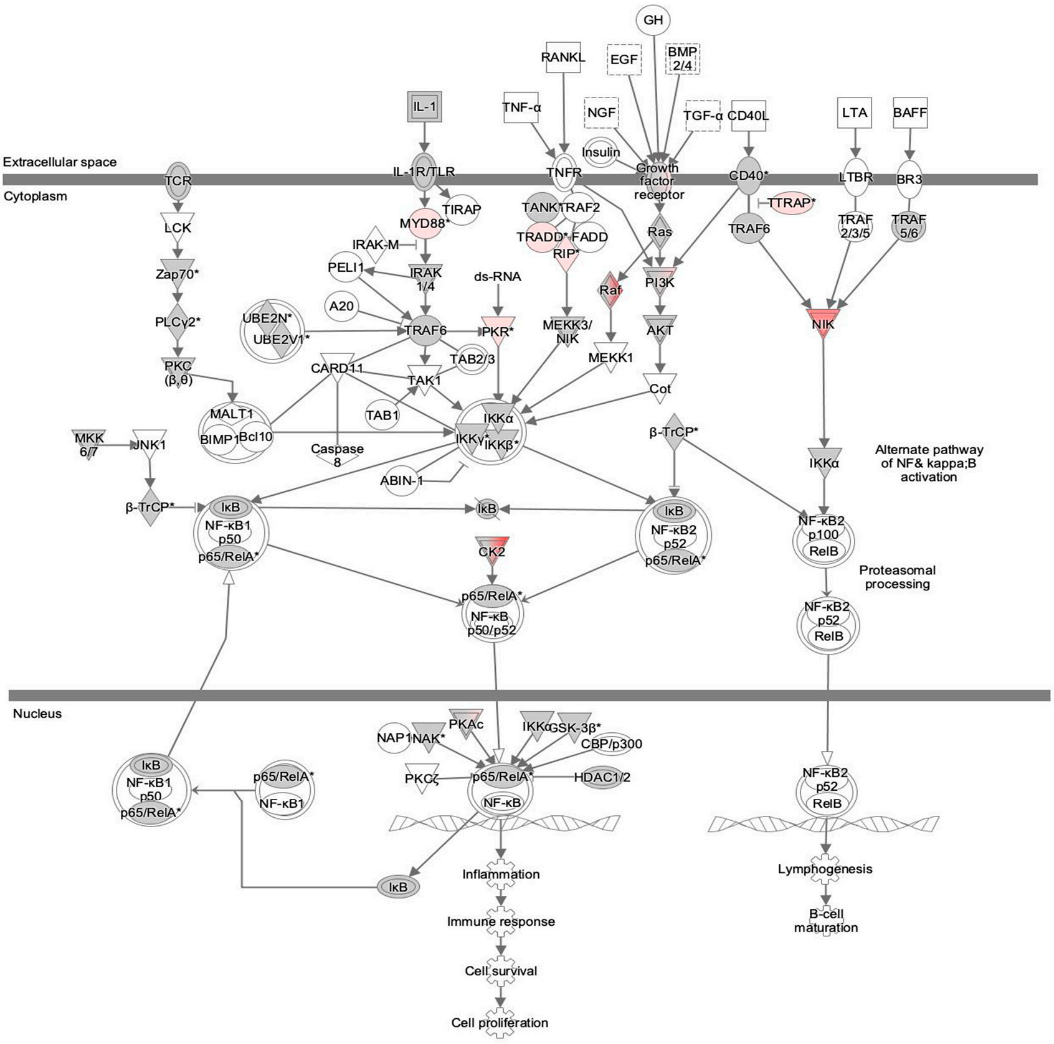

© 2000-2021 QIAGEN. All rights reserved.

Figure S3. Canonical pathway of NF-*κ*B signaling for the HIV infected group compared to uninfected controls. Red symbols represent upregulated proteins. Gray symbols represent unregulated proteins in the dataset. White symbols represent proteins absent in the dataset. Higher color intensity = higher absolute value of fold change expression. This figure was obtained from IPA.

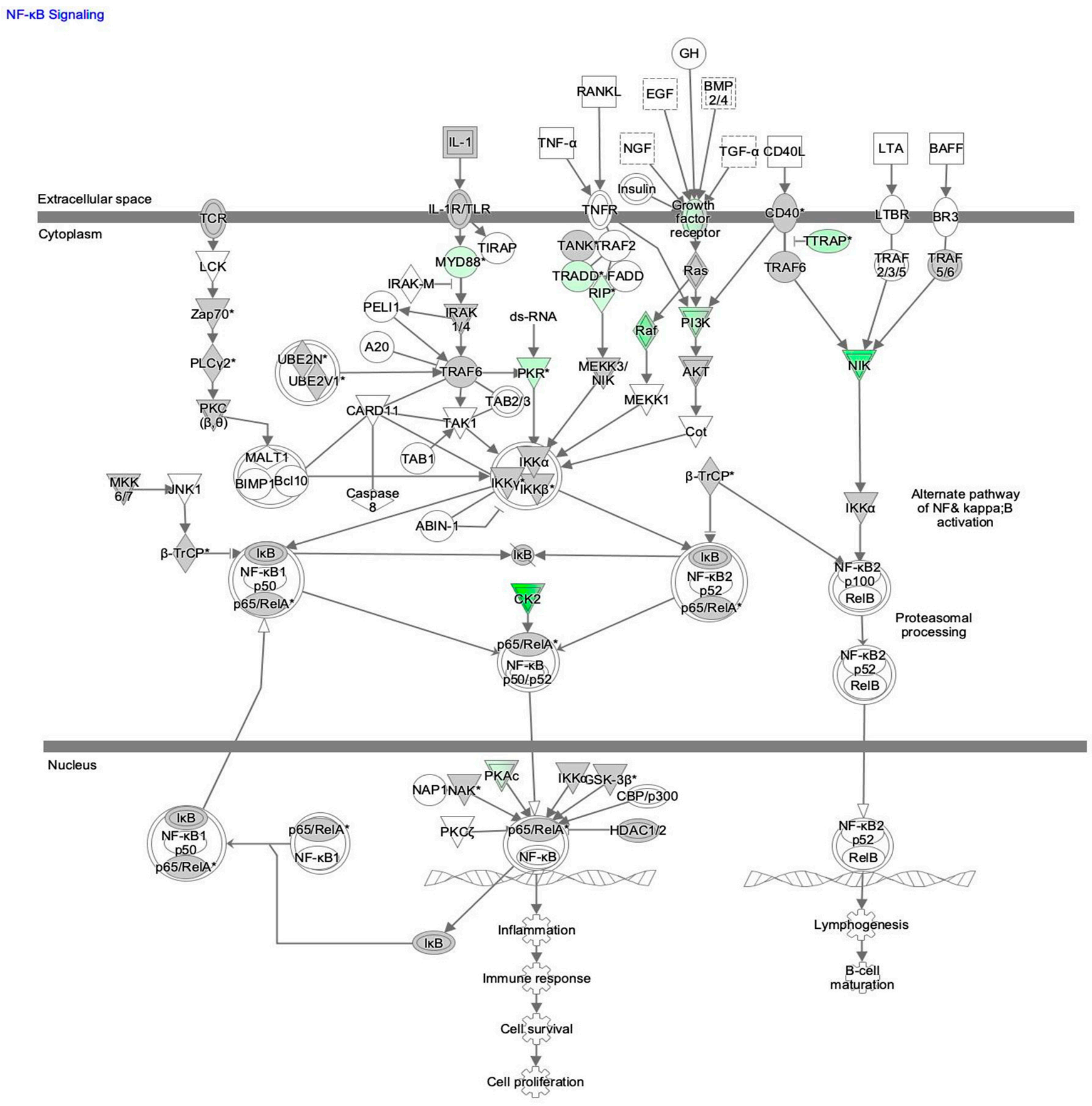

© 2000-2021 QIAGEN. All rights reserved.

Figure S4. Canonical pathway of NF-*κ*B signaling for the HIV + agonist group compared to untreated HIV infected group. Green symbols represent downregulated proteins. Gray symbols represent unregulated proteins in the dataset. White symbols represent proteins absent in the dataset. Higher color intensity = higher absolute value of fold change expression. This figure was obtained from IPA.

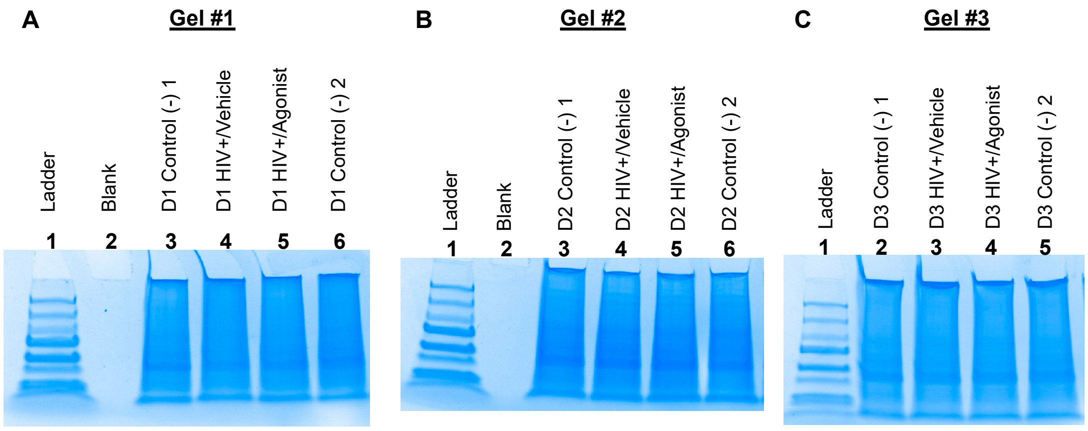

Figure S5. SDS gels. 20 µg of each sample was run in SDS gels and stained with Bio-safe Coomassie G-250 Stain. Each donor was run in a separate gel. Two controls (Uninfected/Vehicle) per donor were run in each gel. **A**. Samples of Donor 1. **B.** Samples of donor 2. **C**. Samples of donor 3.
