# Supplementary material for: Quantitative Proteomics Reveal That CB2R Agonist JWH-133 Downregulates NF-κB Activation, Oxidative Stress, and Lysosomal Exocytosis from HIV-Infected Macrophages
Source: Int J Mol Sci. 2024 Mar 13;25(6):3246. doi: 10.3390/ijms25063246 (PMC10970132; doi:10.3390/ijms25063246)
Supplement: Supplementary file 1 [file ijms-25-03246-s001.zip › Table S3-TMT Tags Description.pdf]

Table S3. TMT Tags Description. A TMT10-plex kit was used to run each of the 9 samples + 1 pool of uninfected/vehicle controls from all donors.

TMT Label	Sample ID	Category
126	*Internal Control – Pool of controls*	*Pool: Uninfected/Vehicle*
127N	D1	(Control)Uninfected/Vehicle
127C	D2	(Control)Uninfected/Vehicle
128N	D3	(Control)Uninfected/Vehicle
128C	D1	HIV+/Vehicle
129N	D2	HIV+/Vehicle
129C	D3	HIV+/Vehicle
130N	D1	HIV+/Agonist
130C	D2	HIV+/Agonist
131	D3	HIV+/Agonist
